# Therapeutic perspectives in food allergy

**DOI:** 10.1186/s12967-020-02466-x

**Published:** 2020-08-05

**Authors:** Francesco Marcucci, Chiara Isidori, Alberto Argentiero, Cosimo Neglia, Susanna Esposito

**Affiliations:** 1 Pediatric Clinic, Department of Surgical and Biomedical Sciences, Università degli Studi di Perugia, Perugia, Italy; 2 Pietro Barilla Children’s Hospital, Department of Medicine and Surgery, University of Parma, Via Gramsci 14, 43126 Parma, Italy

**Keywords:** Key-words, Anaphylaxis, Epicutaneous immunotherapy, Food allergy, Oral immunotherapy, Subcutaneous immunotherapy

## Abstract

**Background:**

In the last twenty years, several studies have been conducted in the search for new therapeutic strategies in patients with food allergy; in particular, after the failure of injection immunotherapy, three different routes of administration, oral immunotherapy (OIT), sublingual immunotherapy (SLIT), and epicutaneous immunotherapy (EPIT), have been tested. The aim of this manuscript is to review OIT, SLIT, and EPIT clinical trials on food allergies and to suggest advantages and limits of the different routes of immunotherapy administration.

**Main body:**

Of the three different routes of immunotherapy used in the treatment of food allergy, OIT is, at present, the only one actually able to induce an increase in tolerance in the majority of patients. However, its use is affected by serious secondary effects, such as major abdominal symptoms and anaphylaxis. The combination with omalizumab reduces the percentage of serious side effects. There are not many studies with SLIT for food allergy, but they have nevertheless shown that it is possible to obtain an increase in tolerance; however, this increase is modest in comparison with that obtained by OIT. EPIT, performed through the diffusion of allergens on intact skin, is the most recent form of immunotherapy. Although there are many works on EPIT carried out in laboratory animals, only few clinical studies have been published in humans. EPIT, unlike OIT and SLIT, is not responsible for systemic secondary effects such as anaphylaxis and eosinophilic oesophagitis but only for local and mild effects in areas where the devices are applied. Moreover, EPIT is characterized by high patient adherence.

**Conclusion:**

OIT seems to have a prevalent application in patients who do not report previous symptoms of systemic or gastroenteric anaphylaxis, while SLIT and EPIT, in particular, could be more preferentially used in patients with a risk of anaphylaxis.

## Background

Food allergy is a continually increasing public health problem affecting millions of adults and children, with a current prevalence of 5% and 10%, respectively [1, 2]. The symptoms that occur following the ingestion of the food may be mild, characterized only by skin itching and hives, or severe, such as anaphylaxis. Several factors influence the extent of the symptomatology, as the amount of food ingested, the presence of asthma, physical exercise, concomitant infectious pathology and the degree of sensitization [3]. Currently, due to limited therapeutic possibilities, the optimal recommendation in presence of food allergy remains a careful elimination diet, which is not always easy to execute, and in patients at risk of anaphylaxis, the availability of adrenaline self-injectors [4, 5].

In the last 20 years, several clinical studies have been conducted in the search for new therapeutic strategies in patients with food allergy. After the failure of injection immunotherapy, three different routes of administration, oral immunotherapy (OIT), sublingual immunotherapy (SLIT), and epicutaneous immunotherapy (EPIT), have been tested [6]. The purpose and goals of this manuscript is to review OIT, SLIT, and EPIT clinical trials on food allergies and to suggest advantages and limits of the different routes of immunotherapy administration.

## Oral immunotherapy

OIT essentially consists of the execution of a food challenge (FC) to assess the tolerating threshold. A minimum initial dose of a few milligrams is administered and then increased in 1–2 days, followed by a scaled dose increase every 1–2 weeks for 6 months [6]. This phase is followed by a maintenance period (i.e., a period without increase in dose) for more than 1 year at home (in some cases up to 3–5 yrs) and upon maintenance the acquired tolerance is evaluated by another FC. Administration of OIT is almost for 2 years. After therapy has been stopped and after a diet for 1–2 weeks, a FC is repeated to verify sustained unresponsiveness [6]. OIT use is affected by serious secondary effects, such as major abdominal symptoms and anaphylaxis [6]. Most patients experience allergic reactions and, although generally mild, severe reactions have occurred. Moreover, long-term adherence is unclear, which rises concerns given the low rates of long-term tolerance induction [6]. Another negative aspect is the loss of tolerance acquired one or more weeks after the end of therapy in the majority of patients who tend to discontinue the therapeutic dose over time. OIT has been performed using several types of food, although cow’s milk, peanut and egg are the food most frequently treated with this approach.

Regarding cow’s milk allergy, the first studies that indicated how the oral administration of small, increasing amounts of a causal food could induce tolerance in cow’s milk allergic patients were published by Patriarca et al. in 1998 (age range, 3–55 years) [7], Meglio et al. in 2004 (age range, 6–17 years) [8] and Longo et al. in 2008 (age range, 5–17 yrs) [9]. These open-label studies were followed by several randomized controlled trials that confirmed a significant increase in tolerance up to 5.1–8 grams of cow’s milk in more than 70% of patients after 23–60 weeks of treatment [10–13]. Some of these studies have shown that the remaining 30% of non-responders discontinued treatment because of major gastrointestinal symptoms or anaphylaxis, which were prevalent in the initial phase of escalation and in the maintenance phase [10, 11]. Other studies reported that 60% of responders did not maintain sustained unresponsiveness after 6–8 weeks of stopping treatment [12, 13]. It is possible that the ones achieved sustained unresponsiveness just have naturally outgrown their allergy.

Regarding peanut allergy, in one of the first studies conducted in the USA in 2009, the authors showed an increase in tolerance up to 3.9 grams of peanuts in 24/29 (82.8%) peanut allergic patients [14]. Subsequent randomized controlled trials confirmed that OIT is able to induce an increase in tolerance up to 5 grams of peanuts in 60% of the subjects treated. However, only 50% of these patients maintained sustained unresponsiveness after treatment suspension [15, 16]. On the contrary, a recent study performed in children of preschool age reported that 29/32 (90.6%) maintained sustained unresponsiveness after treatment [17]. This result suggested the presence of a suitable immunological window to acquire a permanent tolerance in this age group. Interestingly, a recent phase 3 trial of OIT in children and adolescents who were highly allergic to peanut showed that treatment with a peanut-derived investigational biologic oral immunotherapy drug (AR101) resulted in higher doses of peanut protein that could be ingested without dose-limiting symptoms and in lower symptom severity during peanut exposure at the exit food challenge than placebo [18]. Differences between studies can be explained by differences in the study population, the sample size and the study outcomes [15–18].

Regarding egg allergy, after some successful open-label work in children 3–13 years old [19, 20], a controlled OIT study in children 5–15 years old showed that after 22 months of treatment, 70% of egg-allergic patients could tolerate up to 10 grams of powdered food, but in this case, only 28% maintained sustained unresponsiveness after 8 weeks from the end of the therapy [21]. At 30 months and 36 months, all children who had passed the FC at 24 months were consuming egg [21].

### Polysensitized patients or patients allergic to other foods

OIT was used successfully in polysensitized patients, simultaneously administering up to 4 grams of each food, without detecting substantial differences in the acquisition of tolerance and in adverse secondary effects [22].

As there is limited data on the sustainability of desensitization of multifood-OIT [23, 24], Andorf et al. conducted a multisite multifood-OIT study to compare the efficacy of successful desensitization with sustained dosing vs discontinued dosing after multifood-OIT [25]. Results suggested that sustained desensitization after multi-OIT best occurs through continued maintenance OIT dosing of either 300 mg or 1 g of each food allergen as opposed to discontinuation of multi-OIT.

### Association of oral immunotherapy and omalizumab

Although omalizumab (anti-IgE) is currently only approved for the treatment of asthma and chronic idiopathic urticaria, it has also been studied as an off-label treatment for numerous allergic conditions, including use as an adjunct to allergen immunotherapy in the treatment of food allergy. OIT supplemented by omalizumab promotes allergen desensitization “through an initial omalizumab-dependent step that acutely depletes allergen-reactive T cells, followed by an increase in allergen-specific Treg cell activity due to the reversal of their Th2 cell-like programme” [26]. Improved Treg cell function may be a key mechanism by which OIT ameliorates food allergy. Interestingly, Abdel-Gadir et al. showed that an IgE-depleting antibody also led to the depletion of T cells [26], whereas other authors suggested that omalizumab may not influence T cell responses [27].

An open-label study investigating the association of OIT with the humanized anti-IgE antibody omalizumab showed that in only 8 weeks, 12/13 (92.3%) patients could tolerate 4 grams of peanuts [28]. All 12 subjects continued on 4000 mg peanut flour per day and subsequently tolerated a challenge with 8000 mg peanut flour (equivalent to about 20 peanuts), or 160 to 400 times the dose tolerated before desensitization. In subjects allergic to milk, the combination of OIT and omalizumab led to 9/11 (81.8%) patients being able to tolerate 1 g on a single day of protein therapy, although two of these patients received interrupted therapy because of abdominal pain and anaphylaxis, respectively [29]. A comparison study between patients treated with OIT in association with omalizumab and patients treated with OIT alone reported rather disappointing results, particularly regarding the lack of sustained unresponsiveness [30]. Unlike in a recent controlled association study, while 23/29 (79.3%) patients tolerated 4 grams of peanuts in the group of subjects treated with 12 weeks of omalizumab therapy, in the placebo group, only 1/8 (12.5%) was tolerant [31]. In the available studies, the combination with omalizumab reduced the percentage of serious side effects [28–31].

Considering overall the available studies, different methods, different populations, and different endpoints cannot permit to draw any conclusion on the efficacy of omalizumab as adjunct therapy to OIT.

### Association of oral immunotherapy and probiotics

Studies on SLIT and EPIT for allergic rhinitis using novel combinations of allergen together with bacterial adjuvants or Toll-like receptor ligands have reported enhanced tolerogenic effect [32]. For this reason, Tang et al. postulated that a combined immunotherapy approach incorporating a probiotic bacterial adjuvant together with allergen OIT might offer an effective treatment for food allergy [32]. In their controlled study of the association of OIT plus *Lactobacillus rhamnosus* has been successfully conducted, with 26/29 (89.7%) patients being tolerant at the end of the study vs 2/28 (7.1%) patients in the placebo group, and after 2 weeks, 23/28 (82.1%) maintained sustained unresponsiveness [32]. However, there are no subsequent confirmatory studies,.

## Sublingual immunotherapy

SLIT involves the administration of small drops of allergen extract (micrograms to milligrams) under the tongue, which is then eventually spit or swallowed [33]. Doses are approximately 1000-times less than OIT doses. The secondary effects produced by SLIT are mainly characterized by itching and oropharyngeal irritation. SLIT studies, already widely used for the treatment of allergic airway diseases, are not numerous compared to studies conducted with OIT. The study protocols initially used only a few micrograms of allergen, which were increased more gradually with prolongation of therapy times [12, 16, 34]. Research carried out in peanut allergic patients used a variable maintenance dose, in most cases from 2.5 to 3.7 mg of allergen, and a treatment time of approximately 13 months [16, 34].

A controlled study comparing OIT in peanut allergic patients clearly showed that the 22-fold increase in tolerance with SLIT was modest compared to the 141-fold increase with OIT [12]. On the other hand, a study with a high number of patients showed that SLIT was safer than OIT [35]. Secondary effects were not frequent; they were mostly oropharyngeal, although systemic adverse reactions have been reported in the majority of the studies. Relevant data were from a comparative study conducted in 30 milk allergic patients: 10 received only SLIT, 10 received SLIT plus low-dose OIT and 10 received SLIT plus high-dose OIT [36]. This study showed that 8/10 (80%) patients treated with SLIT followed by high-dose OIT exceeded the predicted FC, 6/10 (60%) patients treated with SLIT followed by low-dose OIT exceeded the predicted FC, and only 1/10 (10%) subjects treated with SLIT alone exceeded the predicted FC. However, patients treated with OIT had more systemic side effects, and 6/15 (40%) subjects of the first two groups who passed the FC did not maintain sustained unresponsiveness.

Recently, children with peanut allergy aged 1 to 11 years underwent extended maintenance SLIT with 2 mg/day peanut protein for up to 5 years [37]. Subjects with peanut skin test wheals of less than 5 mm and peanut-specific IgE levels of less than 15 kU/L were allowed to discontinue therapy early. SLIT was assessed through a double-blind, placebo-controlled food challenge with up to 5000 mg of peanut protein after completion of SLIT dosing. Results showed that extended-therapy peanut SLIT provided clinically meaningful desensitization in 67% of children with peanut allergy that was balanced with ease of administration and a favourable safety profile [37].

## Epicutaneous immunotherapy

EPIT was originally tested with allergen administration on scarified skin in order to reduce allergic symptoms [38]. In available literature EPIT showed a more favourable safety and elevated adherence compared to OIT and SLIT: it was well-tolerated and without severe adverse events, with local reactions more frequent, but with no significant difference, in comparison with those who received placebo.

More recently, EPIT administration, carried out in intact skin by means of the diffusion of allergens through the stratum corneum in the epidermis with a VIASKIN occlusion device, was used in the treatment of food allergy in a first study in 2010 in paediatric patients allergic to cow milk proteins [39]. VIASKIN is a technology with proteins loaded into a central polyethylene membrane charged with electrostatic forces. The delivery system creates an occlusive chamber on the skin that generates moisture and releases the proteins from the membrane. The proteins are then absorbed through the skin where they interact with epidermal immune cells. The first work, performed with a dose of 1 µg 3 times per week for 3 months, showed a remarkable increase in tolerance in some of the 10 patients treated with active ingredient [39]. However, this result was not significant when compared to that of the 8 patients treated with placebo. The authors showed that EPIT does not produce systemic adverse events, unlike OIT and SLIT, but only secondary effects near the application site of the occlusion devices on intact skin.

After this first clinical work, three further studies in patients allergic to peanuts were published. Of the three studies, the first randomized controlled trial was performed in 100 patients who received different doses of 20, 100, 250, and 500 µg for 2 weeks [14]. This trial was a safety study that demonstrated the absence of systemic effects and the presence of only local effects. Of the next two controlled trials that both confirmed the high safety of EPIT, the first was a multicentre study performed in a large number of patients (n = 220) who at baseline showed a tolerance of no more than 300 µg of peanuts. After 12 months of therapy with 50–100 or 250 µg of peanuts, the end point was to tolerate 1000 µg or more of protein (approximately 4 peanuts) or a tenfold increase in the initial dose of FC after 12 months of treatment [17]. At the end of therapy, a significant difference was found in the response rate among patients treated with 250 µg of active ingredient or placebo (50% vs 25%) in subjects younger than 12 years. In particular, in the age group of 6 to 11 years (53.6% vs 19.4%), the average tolerated dose increased from 30 to 400 mg of protein. The subjects (n = 171) were treated openly after the first 6 months with 250 µg of protein for 2 years, and the response rate increased, particularly in patients aged 6–12 years, after 1 year to 63.3% and after 2 years to 68.4%, and the reactive cumulative average dose changed from 444 to 1440 µg at the end of 2 years. In the second study carried out in peanut allergic patients, subjects who had a cumulative reactive dose (CRD) ≥ 1044 µg of protein could be admitted to receive a baseline FC. The final end point was to exceed a FC of 5044 µg peanut protein or an increase of at least 10 times the baseline CRD [17]. Compared to subjects treated with placebo (12%), 45% of patients treated for 12 months with 100 µg or peanuts and 48% of patients treated with 250 µg of peanuts showed a significant increase in dose. This increase, similar to that observed in a previous study after 1 year of therapy, was modest.

Recently, the efficacy and adverse events of EPIT with a peanut patch among peanut-allergic children was tested in a phase 3, randomized, double-blind, placebo-controlled trial [39]. The percentage difference in responders at 12 months with the 250-μg peanut-patch therapy vs placebo was 21.7% and was statistically significant, but did not meet the prespecified lower bound of the confidence interval criterion for a positive trial result. The clinical relevance of not meeting this lower bound of the confidence interval with respect to the treatment of peanut-allergic children with EPIT remains to be determined.

## Immunological mechanisms

OIT in peanut allergic patients, in addition to inducing a significant increase in tolerance, significantly reduces prick test values and specific IgEs and increases IgG4 equally [40]. In addition, OIT reduces the levels of interleukin (IL)-5 and IL-13 and the reactivity of basophils. The variations in these parameters are much more evident in preschool-age patients treated with OIT, and sustained unresponsiveness is frequently associated with reduced diameters of prick tests, reduced values of specific IgEs and reduced basal levels and levels in response to FC of Ara h1 and Ara h2 IgE [40].

SLIT, unlike OIT, shows reduced secondary side effects associated with slight decreases in specific IgEs and modest increases in IgG4 [40].

EPIT did not show changes in immunological parameters in the first work published in cow’s milk allergic patients [41, 42], but in peanut allergic patients, it was able to induce change in parameters with an increase in specific IgEs in the first months of therapy followed by a significant reduction and, by contrast, a significant increase in IgG4 from the first month [38].

Very interestingly, studies conducted in laboratory animals show that OIT, SLIT and EPIT induce the activation of different T-reg phenotypes. Unlike OIT and SLIT, which induce T-reg effectors and memory cells, EPIT is also able to induce naive T-reg cells [40]. Moreover, while OIT activates the T-reg-bearing homing receptor for the gastrointestinal system (ccr9) and SLIT for the gastrointestinal and cutaneous system (cla), EPIT is also able to induce the T-reg for the oesophagus (ccr3) and for the lung (ccr4) [40]. These effects on different Treg phenotypes may explain differences in results between OIT, SLIT and EPIT. If data on induction of the most different Treg phenotypes by EPIT are confirmed, EPIT could be the type of immunotherapy with the best effect.

## Conclusions

Different methods (including type and dose of antigens as well as mechanism of action on immune system), different populations, and different endpoints used in the available studies did not permit to draw conclusions on advantages and disadvantages of the three different routes of immunotherapy used in the treatment of food allergy. OIT is, at present, the only one actually able to induce a considerable increase in tolerance in a high percentage of patients receiving this treatment. However, its use is affected by serious secondary effects, such as major abdominal symptoms and anaphylaxis. Another negative aspect is the loss of tolerance acquired one or more weeks after the end of therapy in more than 50% of patients who tend to discontinue the therapeutic dose over time. However, the combination with omalizumab reduced therapy time and the percentage of serious side effects, but it did not appear to change the sustained unresponsiveness at the end of treatment. Further studies are necessary to better understand the advantages and limitations of this combination, also considering differences between patients with mild and severe symptoms.

There are not many studies with SLIT for food allergy, but they have nevertheless shown that, although using only a few micrograms of sublingual allergens, it is possible to obtain a significant increase in tolerance; however, this increase is modest in comparison with that obtained by OIT. On the other hand, the secondary effects produced by SLIT are mainly characterized by itching and oropharyngeal irritation and rarely occur at a distance such as in abdominal pain, auricular itching and symptoms affecting the upper airways. These symptoms and the long period of therapy are, however, responsible for numerous episodes of drop-out. In the studies performed with SLIT, the factors influencing sustained unresponsiveness have not yet been addressed; however, they have been detected in a study of the association of SLIT with low-dose OIT.

EPIT, performed through the diffusion of allergens on intact skin, is the most recent form of immunotherapy used in food allergy. Although there are many works on EPIT carried out in laboratory animals, only four clinical studies have been published in humans. Two studies were conducted in a high number of peanut allergic patients. They showed that EPIT can induce a significant increase in tolerance in more than 50% of patients after 12 months of treatment, particularly in subjects aged between 6 and 11 years. This increase is modest, similar to that obtained with the SLIT, but is important for high safety and compliance. EPIT, unlike OIT and SLIT, is not responsible for systemic secondary effects such as anaphylaxis and eosinophilic oesophagitis but only for local and mild effects in areas where the devices are applied. Moreover, EPIT is characterized by high patient adherence. Work carried out in laboratory animals showed that EPIT, in addition to being active on a gastrointestinal and cutaneous level similar to OIT and SLIT, by inducing the production of naive T-regs is able to exercise its tolerogenic action at the respiratory and oesophageal levels. This result, supported also by the complete absence of anaphylaxis in the studies performed in humans, suggests the possibility of a potential activity system of EPIT.

In conclusion, we believe that OIT could have a prevalent application in patients who do not report previous symptoms of systemic or gastroenteric anaphylaxis, while SLIT and EPIT, in particular, could be more preferentially used for their safety in patients with a risk of anaphylaxis. However, all the immunotherapy methods (OIT, SLIT and EPIT) increase the tolerating threshold but apparently not cure the food allergy.

## Data Availability

The data and materials used are included in the review.

## Abbreviations

CRD: Cumulative reactive dose
EPIT: Epicutaneous immunotherapy
FC: Food challenge
OIT: Oral immunotherapy
SLIT: Sublingual immunotherapy
